# A FRET-based biosensor for the quantification of glucose in culture supernatants of mL scale microbial cultivations

**DOI:** 10.1186/s12934-019-1193-y

**Published:** 2019-08-21

**Authors:** Julia Otten, Niklas Tenhaef, Roman P. Jansen, Johannes Döbber, Lisa Jungbluth, Stephan Noack, Marco Oldiges, Wolfgang Wiechert, Martina Pohl

**Affiliations:** IBG-1: Biotechnology, Forschungszentrum Jülich GmbH, 52425 Jülich, Germany

**Keywords:** Sensor beads, Online glucose measurement, Micro cultivation, Glucose binding protein, mTurquoise2, Venus, BioLector

## Abstract

**Background:**

In most microbial cultivations d-glucose is the main carbon and energy source. However, quantification of d-glucose especially in small scale is still challenging. Therefore, we developed a FRET-based glucose biosensor, which can be applied in microbioreactor-based cultivations. This sensor consists of a glucose binding protein sandwiched between two fluorescent proteins, constituting a FRET pair. Upon d-glucose binding the sensor undergoes a conformational change which is translated into a FRET-ratio change.

**Results:**

The selected sensor shows an apparent K_d_ below 1.5 mM d-glucose and a very high sensitivity of up to 70% FRET-ratio change between the unbound and the glucose-saturated state. The soluble sensor was successfully applied online to monitor the glucose concentration in an *Escherichia coli* culture. Additionally, this sensor was utilized in an at-line process for a *Corynebacterium glutamicum* culture as an example for a process with cell-specific background (e.g. autofluorescence) and medium-induced quenching. Immobilization of the sensor via HaloTag^®^ enabled purification and covalent immobilization in one step and increased the stability during application, significantly.

**Conclusion:**

A FRET-based glucose sensor was used to quantify d-glucose consumption in microtiter plate based cultivations. To the best of our knowledge, this is the first method reported for online quantification of d-glucose in microtiter plate based cultivations. In comparison to d-glucose analysis via an enzymatic assay and HPLC, the sensor performed equally well, but enabled much faster measurements, which allowed to speed up microbial strain development significantly.

## Background

Although a broad variety of chemical compounds is already produced via microbial cultivation, the development of new processes and strains for the production of, e.g., non-natural high value products using synthetic biology approaches and microbial communities is gathering momentum. In this respect, bioprocess development at small scales is becoming ever more important. Microbioreactors enable the acceleration of process development by increasing the throughput, since cultivation and characterization of multiple strains can be parallelized [1]. However, available microbioreactor approaches still cover a limited number of sensors for online measurements, e.g., dissolved oxygen, biomass, pH, and fluorescence. Additional online signals, for example to measure consumption of the C-source, are highly desirable to estimate substrate uptake rates which are often correlated to productivity.

Even though enzymatic assays are routinely used to quantify d-glucose in samples from microbial cultivations [2, 3], application in small scale is still limited to at-line processes. Therefore, samples from respective cultivations require a multi-step workup procedure to be processed with enzymatic assays. In contrast genetically encoded fluorescence-based sensors could in principle be used online, as cells could co-produce such sensors, eliminating time-consuming sample workup operations. To enable high throughput screening using fluorescence-associated cell sorting (FACS) various fluorescence-based sensor techniques have been developed [4]. One option are Förster resonance energy transfer (FRET)-based biosensors [5, 6], which are available for a broad range of small molecules and are almost exclusively used intracellularly [4, 7]. In general FRET-based biosensors consist of two fluorescent probes (donor and acceptor) fused to a central metabolite binding protein (BP). Under optimal conditions FRET occurs between the two probes upon excitation of the donor, which transfers energy also to the acceptor. As a result, both fluorescent probes show different fluorescence intensities depending on the FRET effect. This effect is in a certain range dependent on the concentration of the metabolite recognized by the central binding protein. Due to the conformational changes of the binding protein, the FRET efficiency is either increased or decreased. Biosensors located in the cytoplasm of cells are limited to transmit qualitative information on concentration changes of the target metabolite inside the cell, since such systems cannot be properly calibrated [8]. In contrast, extracellular applications of such sensors in the fermentation broths of producer cells enables quantitative detection of the target metabolite due to an easier calibration of such systems, as we have recently demonstrated for l-lysine [9].

In the present study, we have developed and successfully applied a FRET-based biosensor to monitor d-glucose as a C-source in milliliter scale microbial cultivation experiments. The biosensors were constructed by fusing a cyan (donor; mTurquoise2) and a yellow (acceptor; Venus) variant of the green fluorescent protein (GFP) to either end of a periplasmic glucose/galactose-binding protein (MglB) from *E. coli.* MglB is highly specific for glucose and galactose [10]. Binding of these sugars results in a conformational change [11], which is translated to changes in distance, orientation, and thus energy transfer between the fluorescent proteins. Our sensor construct is based on a previously described glucose sensor (FLII^12^P-glu600µ) [12, 13], but we used mTurquoise2 and Venus as a FRET pair to reduce the environmental influence on the sensor signal. Especially the fluorescence intensity of mTurquoise2 is reportedly more stable and brighter even at changing pH and ion concentrations [14–16]. Venus also exhibits a reduced sensitivity towards such changes compared to other yellow variants of GFP (YFP, Citrine) [17–19]. Besides the signal intensity, the sensor affinity (K_d_) must be adopted to the concentration range of the tested system and its sensitivity, the FRET-ratio change between the unbound state and fully metabolite-saturated state, must be high enough to detect the signal behind the background of the cellular supernatant.

Both aspects were recently addressed by the creation of a glucose sensor toolbox using different linker sequences [20]. From this toolbox a sensor with a flexible (GGS)_4_ linker sequence between mTurquoise2 and the MglB was selected, which increased the transfer efficiency and results in a sensor with an affinity in the low millimolar range (< 1.5 mM; Additional file 1: Figure S1) and a very high sensitivity (~ 70% FRET-ratio change). The sensor constructs used in this work are based on the glucose sensor no. 2 developed previously [20] (for details see “Methods” section and Additional file 1).

A soluble Glu^[−]^ and the Halo-tagged version of the glucose sensor Glu^[+Halo]^ were tested in different formulations to demonstrate the potential for at-line as well as online quantification in two common platform organisms: *Corynebacterium glutamicum* and *Escherichia coli,* in the typical cultivation media CGXII and M9, respectively. The soluble Glu^[−]^ sensor can be reliably applied for at-line measurements, demonstrating the potential of FRET-based sensors for process development. In addition, we developed a simple strategy to enable purification, immobilization, and, most significantly, also stabilization of the sensor via the HaloTag^®^ system [21, 22], which allowed for the application of the immobilized sensor Glu^[+Halo]^ online under cultivation conditions. To the best of our knowledge this is the first time, a FRET-based biosensor was used for the online detection of glucose in milliliter scale cultivation.

## Methods

### Protein design

The biosensor without HaloTag^®^ (Glu^[−]^) used in this study is based on sensor no. 2 in a recent publication [20] with a modification of the hexahistidine tag (His-tag). In contrast to sensor no. 2 the His-tag was translocated to the C-terminus of the protein via overlap extension PCR [23]. Additionally, the central MglB carries a L238M exchange to reduce the affinity for glucose [24]. The His-tag of the biosensor with HaloTag^®^ (Glu^[+Halo]^) remained at the N-terminus while the sequence for the HaloTag^®^ was fused to the C-terminus via Gibson assembly using NEB Gibson assembly kit [22, 25]. For DNA sequences, protein sequences, and primers see Additional file 1.

### Sensor production and purification

For production of both sensor variants (Glu^[−]^ and Glu^[+Halo]^) chemically competent *E. coli* BL21(DE3) cells were transformed with the plasmids encoding the respective sensor variant [26]. Transformed cells were grown over night on LB-agar plates containing 100 mg mL^−1^ ampicillin. A single colony was used to inoculate 20 mL LB media and grown over night at 37 °C. To inoculate the main culture (1 L) in auto induction medium, 1 mL of this pre-culture was used [27]. Cells were grown for 2 h at 37 °C and additional 70 h at 20 °C at 150 rpm in baffled 2 L flasks (400 mL). Cells were harvested by centrifugation and stored at − 20 °C until further use. For sensor purification 10% (w/v) cells were suspended in buffer (20 mM MOPS, 150 mM NaCl, 20 mM imidazole, pH 7.3) and disrupted via high-pressure homogenization in three passages using an Avestin Emulsiflex–C5 (Avestin Europe GmbH, Mannheim, Germany). Purification was performed via the His-tag using affinity chromatography on Ni–NTA agarose (Qiagen, Hilden, Germany), followed by size exclusion chromatography as previously described [9]. Finally, the sensor was concentrated to 20 µM through ultrafiltration (Amicon Ultra Centrifugal filter, 30 kDa cut-off) (Merck Millipore, Darmstadt, Germany) and stored in 20 mM MOPS buffer, pH 7.3 at − 20 °C.

### Protein determination

Protein concentration of the soluble sensor was measured photometrically using the molar extinction coefficient of Venus (ε_515 nm_ = 92,200 mol^−1^ cm^−1^) [18].

### Calibration/binding isotherms

Binding isotherms and the calibration for the at-line process were recorded in a microtiter plate using 50 µL soluble sensor (2 µM), which was mixed with 50 µL MOPS buffer (20 mM, pH 7.3) containing d-glucose in the range from 0 mM to 1000 mM. The measurements were performed in a microtiter plate spectrofluorometer (M-200 or M-1000, Tecan, Männedorf, Switzerland) at room temperature. Binding isotherms with varying medium content of either CGXII- or M9-medium were recorded with medium concentrations ranging from 2.5 to 90% (v/v) by replacing the respective volume of MOPS buffer. For each measurement the arithmetic average of 10 measurement cycles was calculated [8]. mTurquoise2 was excited at 428 ± 20 nm, the corresponding emission of both FRET partners were recorded at 485 ± 20 nm for mTurquoise2 [14] and 528 ± 20 nm for Venus [18]. The FRET-ratio *R* was calculated as fluorescence intensity of the acceptor divided by the intensity of the donor according to Eq. (1).

1 $$R = \frac{{I_{acceptor} }}{{I_{donor} }}$$

Parameters from the binding isotherms were deduced from fitting the data using the following Eq. (2) [28]:

2 $$R = \frac{{\Delta R_{{}} *\left[ S \right]}}{{K_{d} + \left[ S \right]}} + R_{0}$$

were R_0_ describes the FRET-ratio in absence of d-glucose, ΔR (R_sat_ – R_0_) referring to sensor sensitivity, is the maximum change in FRET-ratio at saturation of the sensor with glucose (R_sat_), and the dissociation constant K_d_, which describes the apparent affinity of the sensor, is deduced at half-maximal saturation from the inflection point of the binding isotherm. The normalized ΔR was determined as ΔR/R_0_*100%. Additionally, the dynamic range of a sensor can be derived from the quasi-linear region in the semi-logarithmic representation of the binding isotherm (see Additional file 1: Figure S1). The binding isotherms were recorded on devices differing in resolution and sensitivity, such as plate readers (Tecan M-1000, Tecan M-200), and the microbioreactor BioLector^®^ (m2p labs, Baesweiler, Germany). As a result the deduced apparent affinity of the sensor varied in the range of 0.4 to 1.5 mM, depending on the utilized device. Therefore, calibrations used for further calculation of the d-glucose concentration in the culture broth were always performed in the same device, under the same conditions as the corresponding experiments.

### Stability measurements

Thermal stability measurements were performed by incubating the sensors Glu^[−]^ (2 µM) and Glu^[+Halo]^(immobilized) in MOPS-buffer (20 mM, pH 7.3) at different temperatures (25 °C, 4 °C, and − 20 °C). Measurements were performed in a Tecan M-200 spectrophotometer. After excitation of the donor mTurquoise2 at λ_ex_ 420 ± 9 nm, emission spectra from λ_em_ 460 nm to 650 nm were recorded regularly to follow a possible decrease of the fluorescence intensity. From these spectra the FRET-ratio at maximum emission of mTurquoise2 and Venus was calculated according to Eq. (1). Additionally, SDS-PAGE was performed using 19.5 µL of the respective sensor sample, 7.5 µL NuPAGE^®^ sample reducing agent (10×) and 3 µL NuPage^®^ SDS sample buffer (4×) (ThermoFischer Nunc, Waltham, MA, USA). We used NuPage^®^ 4–12% Bis–TRIS gels of 1 mm thickness (ThermoFischer Nunc, Waltham, MA, USA) and the PageRuler Plus Prestained Protein Ladder (ThermoFischer Nunc, Waltham, MA, USA) as marker. Gels were run at 200 V for 45 min [29].

Measurements to determine the stability against shaking were performed in a flower plate in the BioLector^®^ (m2p labs, Baesweiler, Germany). Here 50 µL of the sensors Glu^[−]^ (2 µM) and Glu^[+Halo]^(immobilized) were mixed with 750 µL of MOPS buffer (20 mM, pH 7.3) or M9 medium, respectively. Fluorescence emission of mTurquoise2 (λ_em_ = 486 ± 5 nm) and Venus (λ_em_ = 532 ± 5 nm) were measured after excitation at λ_ex_ = 430 ± 5 nm.

### At-line analysis

Calibration of the soluble sensor versions (Glu^[−]^) for the at-line analysis was performed as described above. To mimic process conditions *C. glutamicum* ATCC 13032 was grown over night in CGXII medium at 30 °C [30, 31] containing fructose (20 g L^−1^, 112 mM) as the main C-source. A sample of the cell suspension (15 µL) was diluted with 285 µL MOPS buffer (20 mM, pH 7.3) containing glucose in the concentration range from 0 g L^−1^ to 45 g L^−1^ (250 mM). From these diluted samples 50 µL were mixed with the Glu^[−]^ solution (50 µL, 2 µM) in clear 300  µL micro titer plates (ThermoFischer Nunc, Waltham, MA, USA). Calibration was performed in quadruplet.

The at-line process and analysis were carried out on a customized Tecan Freedom EVO200 robotic (Tecan, Männedorf, Switzerland) pipetting platform with integrated BioLector^®^, centrifuge (Sigma Laborzentrifugen GmbH, Osterode am Harz, Germany), and spectrofluorimeter (Tecan M-200) [3]. For cultivation *C. glutamicum* ATCC 13032 was incubated in 1000 µL CGXII medium containing d-glucose (20 g L^−1^) in 48-well flower plates (m2p-labs GmbH, Baesweiler, Germany) at 1400 rpm and 30 °C. Every hour three wells were sampled and a technical duplicate of 15 µL was used for the at-line d-glucose analysis, respectively. The remaining material was centrifuged to remove cells and the supernatant was stored at 4 °C for comparative offline analysis via HPLC and enzymatic d-glucose analysis as earlier described [2]. During cultivation pH, pO_2_, and biomass formation (measured as scattered light of 620 nm, referred to as “backscatter”) were recorded online by the BioLector^®^.

### Sensor immobilization

Immobilization of the Glu^[+Halo]^ sensor was performed at room temperature. Before use, the Halo-Link^®^ resin (particle size = 45–165 µm, Promega, Mannheim, Germany) was washed twice with MOPS buffer (20 mM, pH 7.3). A suspension of the resin (100 µL) was incubated with 1 mL Glu^[+Halo]^ solution (20 µM) for 1 h in a 1.5 mL Eppendorf tube under constant slow inversion. After centrifugation in a tabletop centrifuge (10 s, 2000×*g*, Sprout Minicentrifuge, Biozym, Hessisch Oldendorf, Germany) the supernatant was removed. Afterwards the resin was washed twice with 20 mM MOPS buffer. The immobilized Glu^[+Halo]^ sensor was stored at 4 °C suspended in MOPS buffer (20% v/v) in the dark. Loading of the beads with the sensor was estimated by comparing the absorption of Venus (λ_ex_ = 515 nm, ε = 92,200 M^−1^ cm^−1^) of the sensor solution before and after immobilization.

### Online analysis

Online analysis of the glucose concentration was performed with the immobilized Glu^[+Halo]^ sensor in a BioLector^®^. *E. coli* K12 MG1655 was cultivated in 750 µL M9 medium (modified from [32]) containing 5 g L^−1^ (28 mM) d-glucose, at 900 rpm and 30 °C. Immobilized biosensor (50 µL of the suspension (20% v/v) in MOPS buffer (20 mM, pH 7.3) was added to one row of wells in a 48-well flower plate (m2p labs, Baesweiler, Germany). Apart from biomass concentration (measured as scattered light of 620 nm, referred to as “backscatter”) also two fluorescent signals (λ_ex_ = 430 ± 5 nm, λ_em_ = 486 ± 5 nm and λ_em_ = 532 ± 5 nm) were recorded in the BioLector^®^. d-Glucose concentration standards for the online calibration were prepared by mixing M9 medium (750 µL) with 20 concentrations ranging from 0 mM to 100 mM (0 g L^−1^ to 18 g L^−1^) d-glucose with 50 µL sensor bead suspension on the same plate. The calibration curve is described by a saturation kinetic equation whose parameters are fitted to the measured calibration data by minimizing the sum of squares (Additional file 1: Figure S9).

Prior to the online d-glucose analysis, we monitored the growth of *E. coli* MG1655 under the described process conditions also in the presence of immobilized Glu^[+Halo]^ in more detail to exclude growth limitations. Therefore additionally to the backscatter and the two fluorescent signals, pH, and pO_2_ were recoded online by the BioLector^®^. The resulting data is shown in Additional file 1: Figure S10.

## Results and discussion

### Sensor characterization

The d-glucose concentration in microbial cultivations typically ranges from 0 to 200 mM, which requires a comparably low affinity of the d-glucose sensor in the lower millimolar range to sense d-glucose depletion. The first soluble sensor variant, Glu^[−]^, studied in this work, shows K_d_-values of 0.4 ± 0.1 mM for d-glucose and a very good sensitivity in buffer, as can be deduced from a FRET ratio change (ΔR) of 75% between the unbound and the bound state (Additional file 1: Figure S1). Thus, the detection range in MOPS buffer is between 0.01 mM to 10 mM (0.0018 g L^−1^ to 1.8 g L^−1^) d-glucose.

**Fig. 1:**
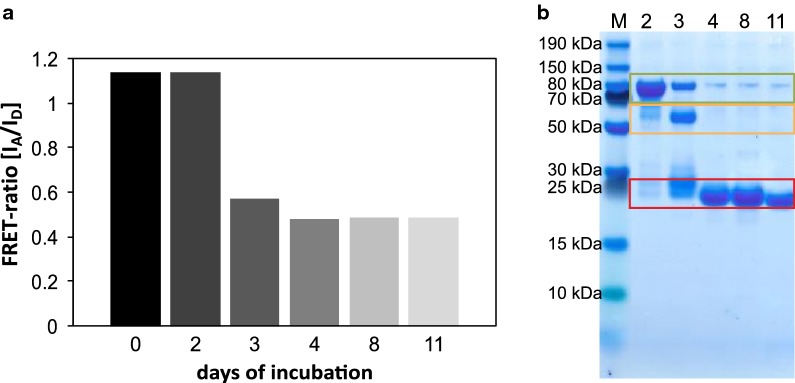
Stability of the Glu^[−]^ sensor (20 µM) in MOPS buffer (20 mM, pH 7.3) at 25 °C. **a** FRET-ratio of the Glu^[−]^ sensor showing clear evidence of degradation after incubation for 3 days. **b** SDS-PAGE analysis of the Glu^[−]^ sensor. Labels above the lanes mark the number of days of incubation. The size of the full-length protein is ~ 90 kDa (green box). After 2 days at 25 °C the sensor starts to degrade into smaller fragments of ~ 60 kDa (orange box, lane 3) and ~ 25–30 kDa (red box). After 4 days (lanes 4–11) the sensor is fully degraded into fragment of ~ 25–30 kDa (for details see text)

Besides high signal intensity the sensor must also be stable under the conditions applied in a microbioreactor, where the biosensor is challenged by temperature and mechanical stress through shaking. First, the thermal stability of the soluble Glu^[−]^ sensor was tested regarding its stability at different temperatures. Whilst the FRET-ratio in the presence and absence of d-glucose remained stable for 21 days of incubation at 4 °C and − 20 °C, respectively, the FRET-ratio was already clearly altered after 3 days at 25 °C (Fig. 1a), which clearly limits the applicability of this sensor at room temperature. Thus, the senor is suitable for lab scale application with cultivation times in the range of 24 to 48 h. SDS-PAGE analysis revealed that the observed instability is caused mainly by an increasing degradation of the fusion protein (Fig. 1b). The biosensor protein degrades into fragments of about 30 kDa (Fig. 1b, red box), which matches the sizes of both FPs as well as the central MglB, respectively. Similar results were also obtained earlier during crystallization attempts of different similar sensors (data not shown). However, the underlying mechanism is still to be elucidated.

Apart from temperature, the sensor must be stable also towards different cultivation media. In order to thoroughly test the application of the novel d-glucose sensor for application with *Corynebacterium glutamicum*, CGXII medium was tested as a typical cultivation medium [30] concerning the influence on the sensor properties. The respective binding isotherm of the Glu^[−]^ sensor recorded in the presence of the culture medium demonstrates a strong influence of CGXII medium on the sensor sensitivity relative to buffer (see Additional file 1: Figure S2). On account of the strong background and quenching of CGXII medium, measurements are only reasonable with CGXII medium diluted with at least 95% buffer (v/v). Additionally, this dilution enables measurements in the presence of *C. glutamicum* cells. Thus, cell separation before at-line measurement of d-glucose is not necessary. Fortunately, the medium did not affect the apparent K_d_ of the sensor variant, indicating that the quenching effect of the medium influences the fluorescent proteins and not the MglB (Additional file 1: Figure S2). The detection limits of 0.01 mM to 10 mM (0.0018 g L^−1^ to 1.8 g L^−1^) d-glucose can, however, be shifted by dilution of the cultivation samples. Dilution by a factor of 40 would enable d-glucose quantification from 0.4 mM to 400 mM (0.072 to 72 g L^−1^), which covers the concentration range of most microbial cultivations.

**Fig. 2:**
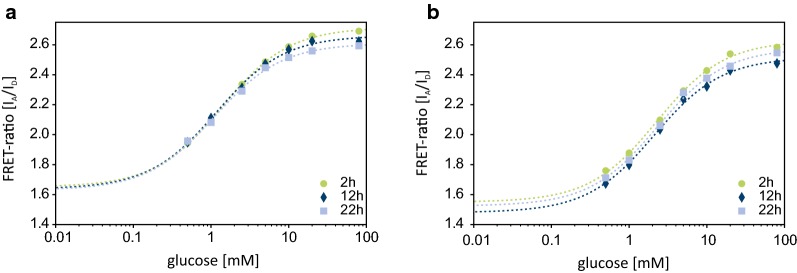
Binding isotherm of the Glu^[−]^ biosensor in either MOPS (20 mM, pH 7.3) (**a**) with CGXII medium (2.5% v/v) and (**b**) without addition of medium without shaking. The FRET-ratio (I_A_/I_D_) was calculated from the emissions of the donor mTurquoise2 at 485 nm (± 20 nm) and acceptor Venus at 528 nm (± 20 nm) after excitation of the donor at 428 nm (± 9 nm) according to Eq. (1). Between measurements the sensor was stored protected from light at 4 °C. The curves (dotted) were fitted to the data according to Eq. (2)

A further prerequisite for the application of such sensors is the reproducibility of calibrations during a typical cultivation experiment, indicating its stability under process conditions. Repeated, comparative calibrations in MOPS buffer in the presence and absence of CGXII medium (2.5% v/v, a dilution of 1:40) showed no significant effect on the apparent affinity (K_d_) and the signal intensity of the Glu^[−]^ sensor. As demonstrated in Fig. 2 the binding isotherms remained stable over the entire experiment, demonstrating that this sensor can be used for repeated measurements.

Besides thermal degradation and media effects, also shaking of the Glu^[−]^ sensor in the Flowerplates^®^ of the BioLector^®^ device turned out to be deleterious, because the emission of both FRET-partners decreased significantly (see Additional file 1: Figure S5). A decrease in both emission intensities indicates a degradation of at least the donor and most likely also the acceptor. Additionally, shaking frequencies > 800 rpm resulted in aggregation of the Glu^[−]^ sensor (data not shown). The aggregated sensor did no longer respond to changes in d-glucose concentration, making the Glu^[−]^ sensor not suitable for an online application in shaken cultures. However, the soluble Glu^[−]^ sensor can be applied in an automated process to measure d-glucose at-line. In such a setting the biosensor stock can easily be stored at 4 °C between measurements, which drastically increases its lifetime. Furthermore, the Glu^[−]^ sensor will not be exposed to shaking, which is beneficial for the sensor stability.

### At-line application of the soluble Glu^[−]^ sensor

With a sufficiently stabile sensor and reproducible calibration at hand, an at-line d-glucose quantification protocol for the widely used production strain *Corynebacterium glutamicum* was established (see Additional file 1: Figure S7). During cultivation of *C. glutamicum* ATCC 13032 in the BioLector^®^, biomass growth, oxygen consumption, and pH changes were monitored online. As described in Methods, three samples were taken every hour by a liquid handling system, diluted, and the d-glucose concentration was measured using the Glu^[−]^ biosensor (see Additional file 1: Figure S8 for calibration). The supernatant of the remaining samples were stored at 4 °C for comparative offline analytics using HPLC and an enzymatic d-glucose assay [2].

**Fig. 3:**
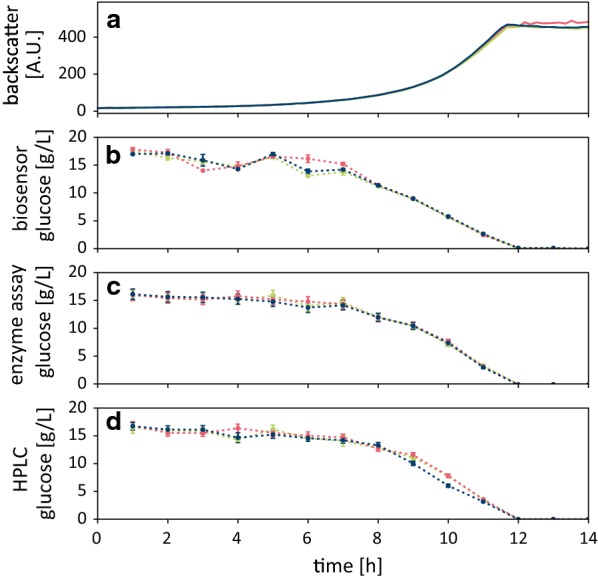
Biomass growth (**a**) and d-glucose consumption of *C. glutamicum* ATCC 13032 in CGXII medium followed via (**b**) the Glu^[−]^ sensor (**c**) enzymatic d-glucose assay, and (**d**) HPLC analysis. While the sensor assay could be performed at-line, enzymatic and HPLC assays were carried out offline after the cultivation was ended. Each curve resembles a biological replicate. The error bars represent the standard deviation (SD) within three technical replicates

As demonstrated in Fig. 3 the Glu^[−]^ sensor assay performed very well and represented the consumption of d-glucose in accordance to HPLC and enzymatic assay analyses. Notably, the measurement was performed in the presence of bacterial cells, using a low amount of sample (15 µL) and a very short incubation time (< 1 min). These properties facilitate a fast measurement process and thus, our workflow allows for quantification of d-glucose in a high number of samples during the runtime of the cultivation process.

Enzymatic assays are routinely used to quantify d-glucose in samples from microbial cultivations [2, 3] and can also be used in an automated at-line setup [33]. However, there are pronounced drawbacks: Firstly, they require previous separation of the bacterial cells, often by means of centrifugation or filtration. This adds complexity to the workflow, is more time-consuming, and requires a complex liquid handling platform. In contrast, the d-glucose sensor assay only needs dilution steps, which can be performed quickly with standard liquid handling operations. Secondly, enzymatic assays involve incubation steps between 10 min [34] and 30 min [2]. While this time-consuming step is unproblematic when large numbers of samples are processed in parallel, it limits the interval of at-line measurements. For example, a previous study demonstrated at-line d-glucose measurements via an enzymatic assay in 80 min intervals [33]. The Glu^[−]^ biosensor, on the other hand, responds immediately to d-glucose in its environment also in the presence of cells, which paves the way to very short measurement cycles and thus increases the density of data.

To the best of our knowledge there is currently no at-line HPLC method established for microbioreactors due to long retention times and sometimes elaborate sample preparations [35]. Whilst sample preparation by filtration could be readily automated, the drawback of measuring only one sample at a time severely hinders the application of at-line HPLC methods in microbioreactor cultivations, which often include multiple parallel cultivations. However, chromatographic methods have the advantage of measuring multiple analytes in one run.

### Immobilization

After having successfully set up an at-line measuring protocol in CGXII medium, we next aimed at the application for online measurements. As demonstrated above, the soluble Glu^[−]^ sensor lacks long term stability at temperatures above 25 °C and is prone to mechanical stress. Mechanical stress cannot be avoided, since agitation is essential for any microbial cultivation to ensure sufficient mixing and, in case of aerobic cultivations, oxygen transfer. Thus, the sensor stability should be improved. Initial immobilization studies via the His-tag failed, because the Co^2+^-chelate bond to the nitrilotriacetic acid-functionalized silica beads (Dynabeads, ThermoScientific) was not stable under process conditions (data not shown). An alternative approach is the immobilization via the HaloTag^®^ [21], which provides covalent immobilization and purification in one step starting also directly from crude cell extracts, as was recently successfully demonstrated for the immobilization of different enzymes [22, 36, 37].

**Fig. 4:**
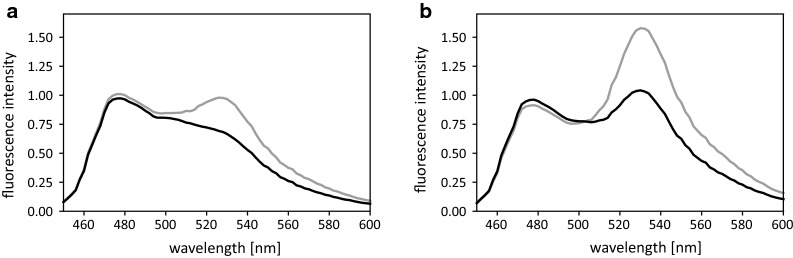
Emission spectra of the Glu^[+Halo]^ sensor (**a**) not immobilized and (**b**) immobilized on HaloLink^®^ resin. Spectra were obtained after excitation of the FRET-donor mTurquoise2 at λ_ex_ = 425 nm (± 9 nm) in the presence (black curve) and absence (grey curve) of 1 M d-glucose. Intensities are normalized to the emission at 485 nm (λ_em_ mTurquoise2)

Fusion of the HaloTag^®^ to the C-terminus of the d-glucose sensor resulted in the sensor Glu^[+Halo]^. This fusion decreased the overall FRET-ratio as well as the ΔR in solution from 75% to almost 40% when compared to the Glu^[−]^ sensor. However, upon immobilization the Glu^[+Halo]^ sensor regains the functionality and high signal intensity (ΔR 74%) of the Glu^[−]^ sensor (for details see Additional file 1: Figure S1). This surprising result can be explained as follows: As we have shown earlier, the FRET efficiency (signal intensity) is strongly influenced by the distance and flexibility of the donor FP mTurquoise2 relative to the central glucose binding protein (MglB) [20]. Due to a similar size of FP and HaloTag^®^ (about 30 kDa), negative steric effects of the C-terminal HaloTag^®^ in a soluble sensor formulation cannot be excluded, which would explain the decrease of the overall transfer efficiency, as shown in the emission spectra (Fig. 4). Here the reduced transfer efficiency is reflected in a decreased emission of the acceptor Venus after excitation of the donor mTurquoise2 (λ_ex_ = 425 ± 9 nm). As the HaloTag^®^ is located at the C-terminus of the sensor, the protein is probably distorted, thereby altering the distance and/or orientation between donor and acceptor. Immobilization on the surface of the Sepharose^®^ beads presumably reduces the interaction of the HaloTag^®^ with the FRET partners resulting in re-established functionality. Remarkably, the affinity (K_d_) of the sensor is not influenced neither by the addition of the tag nor by immobilization. In the soluble formulation, as well as in the immobilized form, the affinity remained in the same range (0.8 mM ± 0.2 mM), indicating that the HaloTag^®^ does not influence the d-glucose binding site (Additional file 1: Figure S1).

Compared to other immobilization techniques for such sensors, such as encapsulation, the site-oriented immobilization via the HaloTag^®^ is superior, as neither the flexibility nor the accessibility of the immobilized sensor is negatively influenced. In a previous study a similar FRET-based d-glucose sensor was encapsulated in silica particles, which significantly reduced the FRET intensity [38]. Furthermore, the biosensor can be directly immobilized from crude cell extract, thereby avoiding laborious and expensive chromatographic protein purification [39].

### Online application of the immobilized Glu^[+Halo]^ sensor

With the Glu^[+Halo]^ sensor covalently immobilized on the surface of Sepharose^®^ beads, the stability of the sensor towards mechanical stress was greatly increased, which was a prerequisite to apply these beads directly in a microbial cultivation (Additional file 1: Figure S6). While the immobilization solves the stability issues, the quenching of CGXII medium remained. Additionally, an increasing concentration of *C. glutamicum* also leads to an increased background due to autofluorescence [40]. To overcome this, the immobilized Glu^[+Halo]^ sensor was tested in M9 medium, a typical cultivation medium for *Escherichia coli* [32]. Despite a reduction of ΔR to 35%, the change in FRET-ratio is distinguishable (see Additional file 1: Figure S3). As a consequence, measurements in M9 medium could be performed with sensor directly in the cultivation, which facilitates an online application.

**Fig. 5:**
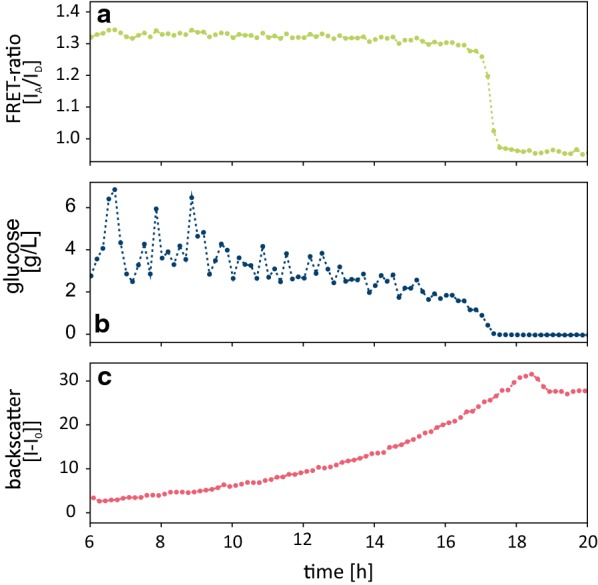
FRET-ratio (**a**), glucose depletion (**b**) and biomass growth (**c**) in an *E. coli* cultivation in M9 medium measured with the FRET-based biosensor immobilized on HaloLink^®^ resin. The FRET-ratio was used to calculate the current d-glucose concentration based on a calibration of the immobilized sensor (see Additional file 1: Figure S9)

The results from online application of the immobilized Glu^[+Halo]^ sensor during a cultivation of *E. coli* K12 MG1655 in M9 medium are shown in Fig. 5. Throughout the cultivation both fluorescent signals of the sensor were monitored by the BioLector^®^ and the FRET-ratio (I_A_/I_D_) was calculated. The extracellular d-glucose concentration was then calculated based on the FRET-ratio and a calibration of the immobilized Glu^[+Halo]^ sensor in the same medium within the same flower plate (see Additional file 1: Figure S9 for the calibration). The consumption of d-glucose could be followed over the entire cultivation experiment (20 h). Here the Glu^[+Halo]^ sensor had a detection range between 0.02 and 2 mM (0.0036 and 0.36 g L^−1^). Consistent with this range, upon depletion of d-glucose (after 18 h), no further change of the FRET-signal could be detected. Even though no d-glucose was left in the medium, the biomass increased further, presumably as a result of an overflow metabolism [41].

The advantage of the online measurement goes in line with the drawback of being limited to a certain measuring window defined by the dynamic range of the sensor. Unlike at-line measurements, where the final d-glucose concentration in the samples can be adopted to the sensor affinity, online measurements require either sensors with a broader dynamic range or different sensors with respective affinities to cover a broader concentration range of the target metabolite. The affinity of a d-glucose sensor can be adjusted either by respective amino acid substitution in the glucose binding proteins [24, 42, 43], by choosing alternative glucose binding proteins with lower affinity [44, 45], or by insertion of linker sequences [9, 20]. As so far no FRET-based sensor with a very broad detection range is known [7], the combination of multiple d-glucose sensors with different affinities and FRET-pairs immobilized on a single carrier could be considered to broaden the detection range.

## Conclusion

We have successfully used a soluble and an immobilized FRET-based d-glucose biosensor to monitor the consumption of d-glucose in small scale microbial cultivations on the example of two common producer strains: *C. glutamicum* and *E. coli*.

We proposed an at-line process using the soluble Glu^[−]^ biosensor. This setup performed well compared to established offline methods like HPLC and enzymatic d-glucose quantification. By using an automated process for sampling and dilution steps, the dynamic range of the Glu^[−]^ sensor was increased by a factor of 40 to 0.4–400 mM (0.072 to 72 g L^−1^) and quenching effects of media components were reduced. The presented sensor retained a very high sensitivity with a FRET-ratio change ~ 60%.

The covalent immobilization of the sensor variant via HaloTag^®^, Glu^[+Halo]^, on Sepharose^®^ beads increased the stability towards mechanical stress while retaining the apparent affinity (~ 0.8 mM) and sensitivity of the soluble Glu^[−]^ sensor. The immobilized sensor was then successfully utilized in a microbioreactor to detect the consumption of d-glucose online. So far, no other direct quantification of d-glucose in small scale cultivation devices is possible. Despite the low detection range of the sensor, the immobilized Glu^[+Halo]^ could be used for the online detection and d-glucose control of typically carbon limited fed-batch experiments in milliliter scale.

To further explore the applicability of the immobilized d-glucose sensor, it can also be used in the proposed at-line process. This would broaden the detectable concentration range and enables its application also in media with a strong background, because the culture supernatant can be diluted. For recycling of the immobilized sensor, magnetic particles would be the best option as those are already available with surface modification for the HaloTag^®^ system. Here, the magnetic retention of beads enables washing and sensor recovery.

In conclusion, FRET-based biosensors are now ready to use for metabolite quantification in culture supernatants. Considering the huge variety of periplasmic binding proteins [7, 42, 46], the range of available FRET pairs [47] and the already available linker toolboxes [9, 20] such biosensors can be tailored for the respective application to promote strain and process development in synthetic biology.

## Supplementary information

**Additional file 1.** Supplementary information.

## Data Availability

The datasets used and/or analyzed during the current study are available from the corresponding author on reasonable request.
