# Supplementary material for: A FRET-based biosensor for the quantification of glucose in culture supernatants of mL scale microbial cultivations
Source: Microb Cell Fact. 2019 Aug 21;18:143. doi: 10.1186/s12934-019-1193-y (PMC6704555; doi:10.1186/s12934-019-1193-y)
Supplement: Supplementary file 1 — Additional file 1. Supplementary information. [file 12934_2019_1193_MOESM1_ESM.docx]

A FRET-based biosensor for the quantification of glucose in culture supernatants of mL scale microbial cultivations

Additional file

Julia Otten^1^, Niklas Tenhaef^1^, Roman Jansen^1^, Johannes Döbber^1^, Lisa Jungbluth^1^, Stephan Noack^1^, Marco Oldiges^1^, Wolfgang Wiechert^1^, Martina Pohl^1^*

^1^Forschungszentrum Jülich GmbH, IBG-1 Biotechnology, D-52415 Jülich, Germany

Contents

[1. Sensor nomenclature 1](#_Toc10016079)

[2. Sequence information: 2](#_Toc10016080)

[3. Primers 5](#_Toc10016081)

[4. Binding isotherms of the novel glucose sensor variants 5](#_Toc10016082)

[5. Influence of cultivation media on the FRET-ratio 6](#_Toc10016083)

[6. Sensor stability 7](#_Toc10016084)

[7. At-line measurement process scheme 9](#_Toc10016085)

[8. Calibrations 9](#_Toc10016086)

# Sensor nomenclature

Table S1: Composition of the sensor used in this study: Positions of the His_6_-tag and HaloTag as well as molecular weights and tested formulation of the sensors Glu^[-]^ and Glu^[+Halo]^.

| sensor | position His_6_-tag | position HaloTag® | formulation | molecular weight |
| --- | --- | --- | --- | --- |
| Glu ^[-]^ | C-terminus | - | soluble | 98,550 g/mol |
| Glu^[+Halo]^ | N-terminus | C-terminus | soluble or immobilized | 128,303 g/mol |

# Sequence information:

Sequences encoding the binding protein (MglB) are depicted green, restriction sites red, mTurquoise2 is colored blue, Venus yellow. The hexahistidine tag (His-tag) is depicted in gray and the HaloTag® is grey underlined. Linker sequences are colored magenta.

Sequence S1: Amino acid sequence encoding the Glu ^[-]^ sensor with N-terminal (relative to MglB) flexible linker ((GGS)_4_), and C-terminal His-tag.

MADTRIGVTIYKAAAMVSKGEELFTGVVPILVELDGDVNGHKFSVSGEGEGDATYGKLTLKFICTTGKLPVPWPTLVTTLSWGVQCFARYPDHMKQHDFFKSAMPEGYVQERTIFFKDDGNYKTRAEVKFEGDTLVNRIELKGIDFKEDGNILGHKLEYNYFSDNVYITADKQKNGIKANFKIRHNIEDGGVQLADHYQQNTPIGDGPVLLPDNHYLSTQSKLSKDPNEKRDHMVLLEFVTAAGITLGMDELYGSGGSGGSGGSGGSPGDNFMSVVRKAIEQDAKAAPDVQLLMNDSQNDQSKQNDQIDVLLAKGVKALAINLVDPAAAGTVIEKARGQNVPVVFFNKEPSRKALDSYDKAYYVGTDSKESGIIQGDLIAKHWAANQGWDLNKDGQIQFVLLKGEPGHPDAEARTTYVIKELNDKGIKTEQLQLDTAMWDTAQAKDKMDAWLSGPNANKIEVVIANNDAMAMGAVEALKAHNKSSIPVFGVDAMPEALALVKSGALAGTVLNDANNQAKATFDLAKNLADGKGAADGTNWKIDNKVVRVPYVGVDKDNLAEFSKKEFVDGGMVSKGEELFTGVVPILVELDGDVNGHKFSVSGEGEGDATYGKLTLKLICTTGKLPVPWPTLVTTLGYGLQCFARYPDHMKQHDFFKSAMPEGYVQERTIFFKDDGNYKTRAEVKFEGDTLVNRIELKGIDFKEDGNILGHKLEYNYNSHNVYITADKQKNGIKANFKIRHNIEDGGVQLADHYQQNTPIGDGPVLLPDNHYLSYQSALSKDPNEKRDHMVLLEFVTAAGITLGMDELYKHHHHHH

Sequence S2: Nucleotide sequence encoding the Glu ^[-]^ sensor with N-terminal (relative to MglB) flexible linker ((GGS)_4_), and C-terminal His-tag.

ATGGCTGATACTCGCATTGGTGTAACAATCTATAAGGCGGCCGCTATGGTGAGCAAGGGCGAGGAGCTGTTCACCGGGGTGGTGCCCATCCTGGTCGAGCTGGACGGCGACGTAAACGGCCACAAGTTCAGCGTGTCCGGCGAGGGCGAGGGCGATGCCACCTACGGCAAGCTGACCCTGAAGTTCATCTGCACCACCGGCAAGCTGCCCGTGCCCTGGCCCACCCTCGTGACCACCCTGTCCTGGGGCGTGCAGTGCTTCGCCCGCTACCCCGACCACATGAAGCAGCACGACTTCTTCAAGTCCGCCATGCCCGAAGGCTACGTCCAGGAGCGCACCATCTTCTTCAAGGACGACGGCAACTACAAGACCCGCGCCGAGGTGAAGTTCGAGGGCGACACCCTGGTGAACCGCATCGAGCTGAAGGGCATCGACTTCAAGGAGGACGGCAACATCCTGGGGCACAAGCTGGAGTACAACTACTTTAGCGACAACGTCTATATCACCGCCGACAAGCAGAAGAACGGCATCAAGGCCAACTTCAAGATCCGCCACAACATCGAGGACGGCGGCGTGCAGCTCGCCGACCACTACCAGCAGAACACCCCCATCGGCGACGGCCCCGTGCTGCTGCCCGACAACCACTACCTGAGCACCCAGTCCAAGCTGAGCAAAGACCCCAACGAGAAGCGCGATCACATGGTCCTGCTGGAGTTCGTGACCGCCGCCGGGATCACTCTCGGCATGGACGAGCTGTACGGATCCGGTGGTTCTGGCGGTTCAGGTGGCTCTGGTGGGTCACCTGGTGATAACTTTATGTCTGTAGTGCGCAAGGCTATTGAGCAAGATGCGAAAGCCGCGCCAGATGTTCAGCTGCTGATGAATGATTCTCAGAATGACCAGTCCAAGCAGAACGATCAGATCGACGTATTGCTGGCGAAAGGGGTGAAGGCACTGGCAATCAACCTCGTTGACCCGGCAGCTGCGGGTACGGTGATTGAGAAAGCGCGTGGGCAAAACGTGCCGGTGGTTTTCTTCAACAAAGAACCGTCTCGTAAGGCGCTGGATAGCTACGACAAAGCCTACTACGTTGGCACTGACTCCAAAGAGTCCGGCATTATTCAAGGCGATTTGATTGCTAAACACTGGGCGGCGAATCAGGGTTGGGATCTGAACAAAGACGGTCAGATTCAGTTCGTACTGCTGAAAGGTGAACCGGGCCATCCGGATGCAGAAGCACGTACCACTTACGTGATTAAAGAATTGAACGATAAAGGCATCAAAACTGAACAGTTACAGTTAGATACCGCAATGTGGGACACCGCTCAGGCGAAAGATAAGATGGACGCCTGGCTGTCTGGCCCGAACGCCAACAAAATCGAAGTGGTTATCGCCAACAACGATGCGATGGCAATGGGCGCGGTTGAAGCGCTGAAAGCACACAACAAGTCCAGCATTCCGGTGTTTGGCGTCGATGCGATGCCAGAAGCGCTGGCGCTGGTGAAATCCGGTGCACTGGCGGGCACCGTACTGAACGATGCTAACAACCAGGCGAAAGCGACCTTTGATCTGGCGAAAAACCTGGCCGATGGTAAAGGTGCGGCTGATGGCACCAACTGGAAAATCGACAACAAAGTGGTCCGCGTACCTTATGTTGGCGTAGATAAAGACAACCTGGCTGAGTTCAGCAAGAAAGAATTCGTCGACGGTGGAATGGTGAGCAAGGGCGAGGAGCTGTTCACCGGGGTGGTGCCCATCCTGGTCGAGCTGGACGGCGACGTAAACGGCCACAAGTTCAGCGTGTCCGGCGAGGGCGAGGGCGATGCCACCTACGGCAAGCTGACCCTGAAGCTGATCTGCACCACCGGCAAGCTGCCCGTGCCCTGGCCCACCCTCGTGACCACCCTGGGCTACGGCCTGCAGTGCTTCGCCCGCTACCCCGACCACATGAAGCAGCACGACTTCTTCAAGTCCGCCATGCCCGAAGGCTACGTCCAGGAGCGCACCATCTTCTTCAAGGACGACGGCAACTACAAGACCCGCGCCGAGGTGAAGTTCGAGGGCGACACCCTGGTGAACCGCATCGAGCTGAAGGGCATCGACTTCAAGGAGGACGGCAACATCCTGGGGCACAAGCTGGAGTACAACTACAACAGCCACAACGTCTATATCACCGCCGACAAGCAGAAGAACGGCATCAAGGCCAACTTCAAGATCCGCCACAACATCGAGGACGGCGGCGTGCAGCTCGCCGACCACTACCAGCAGAACACCCCCATCGGCGACGGCCCCGTGCTGCTGCCCGACAACCACTACCTGAGCTACCAGTCCGCCCTGAGCAAAGACCCCAACGAGAAGCGCGATCACATGGTCCTGCTGGAGTTCGTGACCGCCGCCGGGATCACTCTCGGCATGGACGAGCTGTACAAGCATCATCACCACCATCAC

Sequence S3: Amino acid sequence encoding the Glu^[+Halo]^ sensor with C-terminal Halo-tag, N-terminal (relative to MglB) flexible linker ((GGS)_4_), and N-terminal His-tag.

MRGSHHHHHHGMASMTGGQQMGRDLYDDDDKEPGRADTRIGVTIYKAAAMVSKGEELFTGVVPILVELDGDVNGHKFSVSGEGEGDATYGKLTLKFICTTGKLPVPWPTLVTTLSWGVQCFARYPDHMKQHDFFKSAMPEGYVQERTIFFKDDGNYKTRAEVKFEGDTLVNRIELKGIDFKEDGNILGHKLEYNYFSDNVYITADKQKNGIKANFKIRHNIEDGGVQLADHYQQNTPIGDGPVLLPDNHYLSTQSKLSKDPNEKRDHMVLLEFVTAAGITLGMDELYGSGGSGGSGGSGGSPGDNFMSVVRKAIEQDAKAAPDVQLLMNDSQNDQSKQNDQIDVLLAKGVKALAINLVDPAAAGTVIEKARGQNVPVVFFNKEPSRKALDSYDKAYYVGTDSKESGIIQGDLIAKHWAANQGWDLNKDGQIQFVLLKGEPGHPDAEARTTYVIKELNDKGIKTEQLQLDTAMWDTAQAKDKMDAWLSGPNANKIEVVIANNDAMAMGAVEALKAHNKSSIPVFGVDAMPEALALVKSGALAGTVLNDANNQAKATFDLAKNLADGKGAADGTNWKIDNKVVRVPYVGVDKDNLAEFSKKEFVDGGMVSKGEELFTGVVPILVELDGDVNGHKFSVSGEGEGDATYGKLTLKLICTTGKLPVPWPTLVTTLGYGLQCFARYPDHMKQHDFFKSAMPEGYVQERTIFFKDDGNYKTRAEVKFEGDTLVNRIELKGIDFKEDGNILGHKLEYNYNSHNVYITADKQKNGIKANFKIRHNIEDGGVQLADHYQQNTPIGDGPVLLPDNHYLSYQSALSKDPNEKRDHMVLLEFVTAAGITLGMDELYKLAEAAAKEAAAKEAAAKEAAAKAAAAEIGTGFPFDPHYVEVLGERMHYVDVGPRDGTPVLFLHGNPTSSYVWRNIIPHVAPTHRCIAPDLIGMGKSDKPDLGYFFDDHVRFMDAFIEALGLEEVVLVIHDWGSALGFHWAKRNPERVKGIAFMEFIRPIPTWDEWPEFARETFQAFRTTDVGRKLIIDQNVFIEGTLPMGVVRPLTEVEMDHYREPFLNPVDREPLWRFPNELPIAGEPANIVALVEEYMDWLHQSPVPKLLFWGTPGVLIPPAEAARLAKSLPNCKAVDIGPGLNLLQEDNPDLIGSEIARWLSTLEISG

Sequence S4: Nucleotide sequence encoding the Glu ^[+Halo]^ sensor with C-terminal Halo-tag, N-terminal (relative to MglB) flexible linker ((GGS)_4_), and N-terminal His-tag.

ATGCGGGGTTCTCATCATCATCATCATCATGGTATGGCTAGCATGACTGGTGGACAGCAAATGGGTCGGGATCTGTACGACGATGACGATAAGGAGCCGGGCCGCGCTGATACTCGCATTGGTGTAACAATCTATAAGGCGGCCGCTATGGTGAGCAAGGGCGAGGAGCTGTTCACCGGGGTGGTGCCCATCCTGGTCGAGCTGGACGGCGACGTAAACGGCCACAAGTTCAGCGTGTCCGGCGAGGGCGAGGGCGATGCCACCTACGGCAAGCTGACCCTGAAGTTCATCTGCACCACCGGCAAGCTGCCCGTGCCCTGGCCCACCCTCGTGACCACCCTGTCCTGGGGCGTGCAGTGCTTCGCCCGCTACCCCGACCACATGAAGCAGCACGACTTCTTCAAGTCCGCCATGCCCGAAGGCTACGTCCAGGAGCGCACCATCTTCTTCAAGGACGACGGCAACTACAAGACCCGCGCCGAGGTGAAGTTCGAGGGCGACACCCTGGTGAACCGCATCGAGCTGAAGGGCATCGACTTCAAGGAGGACGGCAACATCCTGGGGCACAAGCTGGAGTACAACTACTTTAGCGACAACGTCTATATCACCGCCGACAAGCAGAAGAACGGCATCAAGGCCAACTTCAAGATCCGCCACAACATCGAGGACGGCGGCGTGCAGCTCGCCGACCACTACCAGCAGAACACCCCCATCGGCGACGGCCCCGTGCTGCTGCCCGACAACCACTACCTGAGCACCCAGTCCAAGCTGAGCAAAGACCCCAACGAGAAGCGCGATCACATGGTCCTGCTGGAGTTCGTGACCGCCGCCGGGATCACTCTCGGCATGGACGAGCTGTACGGATCCGGTGGTTCTGGCGGTTCAGGTGGCTCTGGTGGGTCACCTGGTGATAACTTTATGTCTGTAGTGCGCAAGGCTATTGAGCAAGATGCGAAAGCCGCGCCAGATGTTCAGCTGCTGATGAATGATTCTCAGAATGACCAGTCCAAGCAGAACGATCAGATCGACGTATTGCTGGCGAAAGGGGTGAAGGCACTGGCAATCAACCTCGTTGACCCGGCAGCTGCGGGTACGGTGATTGAGAAAGCGCGTGGGCAAAACGTGCCGGTGGTTTTCTTCAACAAAGAACCGTCTCGTAAGGCGCTGGATAGCTACGACAAAGCCTACTACGTTGGCACTGACTCCAAAGAGTCCGGCATTATTCAAGGCGATTTGATTGCTAAACACTGGGCGGCGAATCAGGGTTGGGATCTGAACAAAGACGGTCAGATTCAGTTCGTACTGCTGAAAGGTGAACCGGGCCATCCGGATGCAGAAGCACGTACCACTTACGTGATTAAAGAATTGAACGATAAAGGCATCAAAACTGAACAGTTACAGTTAGATACCGCAATGTGGGACACCGCTCAGGCGAAAGATAAGATGGACGCCTGGCTGTCTGGCCCGAACGCCAACAAAATCGAAGTGGTTATCGCCAACAACGATGCGATGGCAATGGGCGCGGTTGAAGCGCTGAAAGCACACAACAAGTCCAGCATTCCGGTGTTTGGCGTCGATGCGATGCCAGAAGCGCTGGCGCTGGTGAAATCCGGTGCACTGGCGGGCACCGTACTGAACGATGCTAACAACCAGGCGAAAGCGACCTTTGATCTGGCGAAAAACCTGGCCGATGGTAAAGGTGCGGCTGATGGCACCAACTGGAAAATCGACAACAAAGTGGTCCGCGTACCTTATGTTGGCGTAGATAAAGACAACCTGGCTGAGTTCAGCAAGAAAGAATTCGTCGACGGTGGAATGGTGAGCAAGGGCGAGGAGCTGTTCACCGGGGTGGTGCCCATCCTGGTCGAGCTGGACGGCGACGTAAACGGCCACAAGTTCAGCGTGTCCGGCGAGGGCGAGGGCGATGCCACCTACGGCAAGCTGACCCTGAAGCTGATCTGCACCACCGGCAAGCTGCCCGTGCCCTGGCCCACCCTCGTGACCACCCTGGGCTACGGCCTGCAGTGCTTCGCCCGCTACCCCGACCACATGAAGCAGCACGACTTCTTCAAGTCCGCCATGCCCGAAGGCTACGTCCAGGAGCGCACCATCTTCTTCAAGGACGACGGCAACTACAAGACCCGCGCCGAGGTGAAGTTCGAGGGCGACACCCTGGTGAACCGCATCGAGCTGAAGGGCATCGACTTCAAGGAGGACGGCAACATCCTGGGGCACAAGCTGGAGTACAACTACAACAGCCACAACGTCTATATCACCGCCGACAAGCAGAAGAACGGCATCAAGGCCAACTTCAAGATCCGCCACAACATCGAGGACGGCGGCGTGCAGCTCGCCGACCACTACCAGCAGAACACCCCCATCGGCGACGGCCCCGTGCTGCTGCCCGACAACCACTACCTGAGCTACCAGTCCGCCCTGAGCAAAGACCCCAACGAGAAGCGCGATCACATGGTCCTGCTGGAGTTCGTGACCGCCGCCGGGATCACTCTCGGCATGGACGAGCTGTACAAGCTTGCAGAAGCAGCGGCCAAAGAAGCTGCGGCCAAAGAGGCAGCCGCGAAAGAAGCAGCGGCGAAAGCGGCCGCGGCAGAAATTGGTACGGGATTTCCGTTTGACCCGCATTATGTGGAGGTTCTGGGTGAACGCATGCACTACGTGGATGTTGGTCCGCGCGATGGCACACCGGTGCTGTTTCTGCATGGTAATCCGACCTCCAGCTATGTTTGGCGCAACATTATTCCGCATGTCGCCCCAACGCATCGCTGTATTGCCCCAGATCTCATTGGCATGGGCAAAAGCGACAAACCGGATTTGGGCTACTTCTTCGACGATCACGTACGGTTTATGGACGCCTTTATCGAGGCTCTGGGACTCGAGGAAGTAGTGCTGGTTATTCATGACTGGGGCTCTGCATTAGGCTTTCACTGGGCTAAACGGAACCCAGAACGCGTCAAGGGGATTGCCTTCATGGAGTTCATCCGTCCGATTCCGACCTGGGATGAATGGCCCGAATTTGCCCGTGAAACCTTTCAGGCGTTTCGTACCACGGATGTTGGCCGTAAGCTCATCATCGACCAAAACGTGTTCATTGAGGGCACTCTTCCCATGGGAGTAGTGCGTCCTTTAACCGAAGTCGAGATGGACCACTATCGCGAACCCTTCCTGAATCCGGTTGATCGCGAACCGCTGTGGCGCTTCCCGAATGAGCTGCCTATTGCTGGTGAACCGGCGAATATCGTGGCACTTGTGGAAGAATACATGGATTGGCTGCATCAGAGTCCAGTCCCTAAGCTGTTGTTTTGGGGTACACCTGGCGTGTTGATTCCGCCTGCAGAAGCTGCTCGCTTAGCGAAAAGCTTGCCCAACTGCAAAGCGGTCGATATTGGGCCAGGTCTGAACCTGTTACAGGAGGATAACCCGGATCTGATCGGGAGTGAAATCGCGCGTTGGCTGTCAACTCTGGAAATCTCGGGTTAA

# Primers

Table S2: Used primers for biosensor modifications. For the Gibson assembly of the Glu^[+Halo]^ sensor with HaloTag, primers 1&2 were used. Primers 3-10 were used for translocation of the His-tag in the Glu^[-]^ sensor from the N-to C-terminus via Overlap Extension PCR (Ex. Ov.).

| primer 1 | CTTTAAGAAGGAGATATACATATGGCAGAAATTGGTACGGGATTTCCGTTTGAC | Gibson |
| --- | --- | --- |
| primer 2 | CAATGCGAGTATCAGCCATCGCGGCCGCTTTCG | Gibson |
| primer 3 | CATCATCACCACCATCACTAAAAGCTTGATCCGGCTGCTAAC | Ex.ov. |
| primer 4 | TTCTTAAAGTTAAACAAAATTATCTAGAGGGAAACCGTTGTGGTCTCCCTATAGTG | Ex.ov. |
| primer 5 | CATCACTAAAAGCTTGATCCGGCTGCTAAC | Ex.ov. |
| primer 6 | GCCATATGTATATCTCCTTCTTAAAGTTAAACAAAATTATC | Ex.ov. |
| primer 7 | GGAGATATACATATGGCTGATACTCGCATTGGTGTAAC | Ex.ov. |
| primer 8 | CTTGTACAGCACCATTCCACCGTCG | Ex.ov. |
| primer 9 | TGATACTCGCATTGGTGTAACAATCTATAAGG | Ex.ov. |
| primer 10 | GTGGTGATGATGCTTGTACAGCACCATTCCACCGTCG | Ex.ov. |

# Binding isotherms of the novel glucose sensor variants


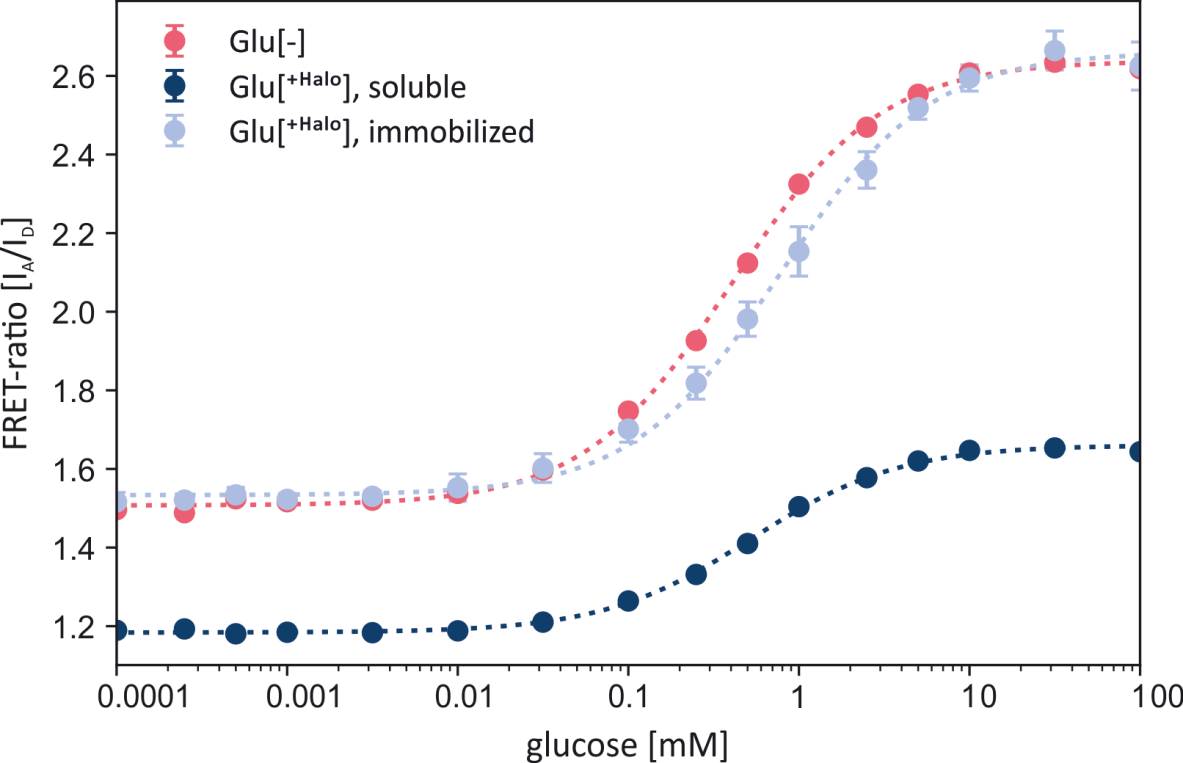


Figure S1: Comparison of binding isotherms of the Glu^[-]^ sensor and the Glu^[+Halo]^ sensor in the non-immobilized form and immobilized on HaloLink Sepharose® beads, respectively, in MOPS buffer (20 mM, pH 7.3). The Glu^[-]^ sensor without HaloTag® shows an affinity (K_d_) of 0.4 ± 0.1 mM glucose and a sensitivity (ΔR) of 75 % (red). For the non-immobilized Glu^[+Halo]^ sensor the sensitivity dropped to 40 % (dark blue). Immobilization of Glu^[+Halo]^ sensor restores the functionality similar to the Glu^[-]^ sensor without HaloTag® (0.8 ± 0.2 mM, 74 % ΔR) (light blue).

# Influence of cultivation media on the FRET-ratio


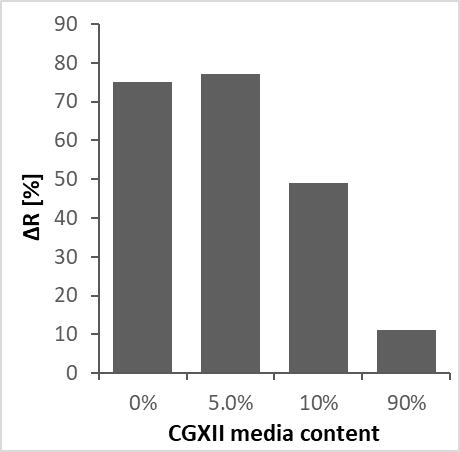

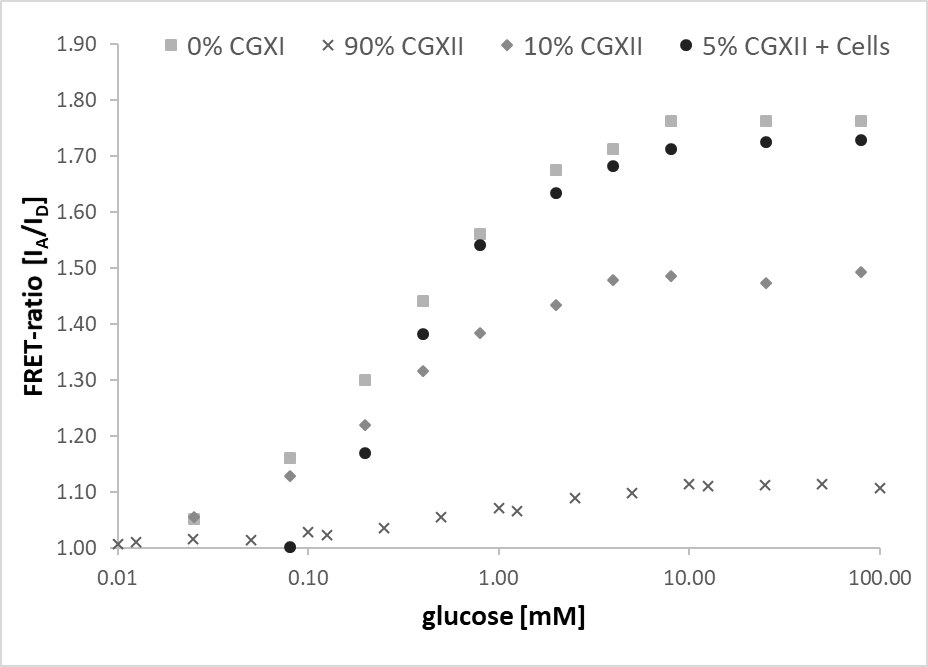


Figure S2: Binding isotherms of the Glu ^[-]^ sensor with varying volumes of CGXII medium (left). In the right figure, values were normalized to the respective R_0_ values. The FRET-ratio change ΔR was highest (75%) in MOPS buffer (20 mM, pH 7.3) in the absence of medium (0 vol%). Addition of 5 vol% medium did not change the sensitivity of the Glu^[-]^ sensor (ΔR = 74%). Addition of larger medium fractions decreases the sensitivity progressively. The affinity (Kd) was not influences and remained in the range of 0.4 ± 0.1 mM.


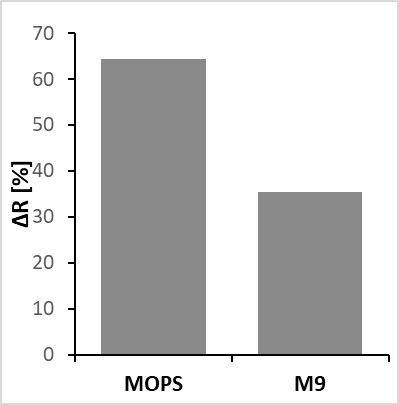


Figure S3: FRET-ratio change of the immobilized Glu^[+Halo-]^ sensor (on HaloLink Sepharose® resin). The addition of 90 vol% M9 medium reduced ΔR from 64 % in MOPS buffer (20 mM, pH 7.3) to 35 %.

# Sensor stability


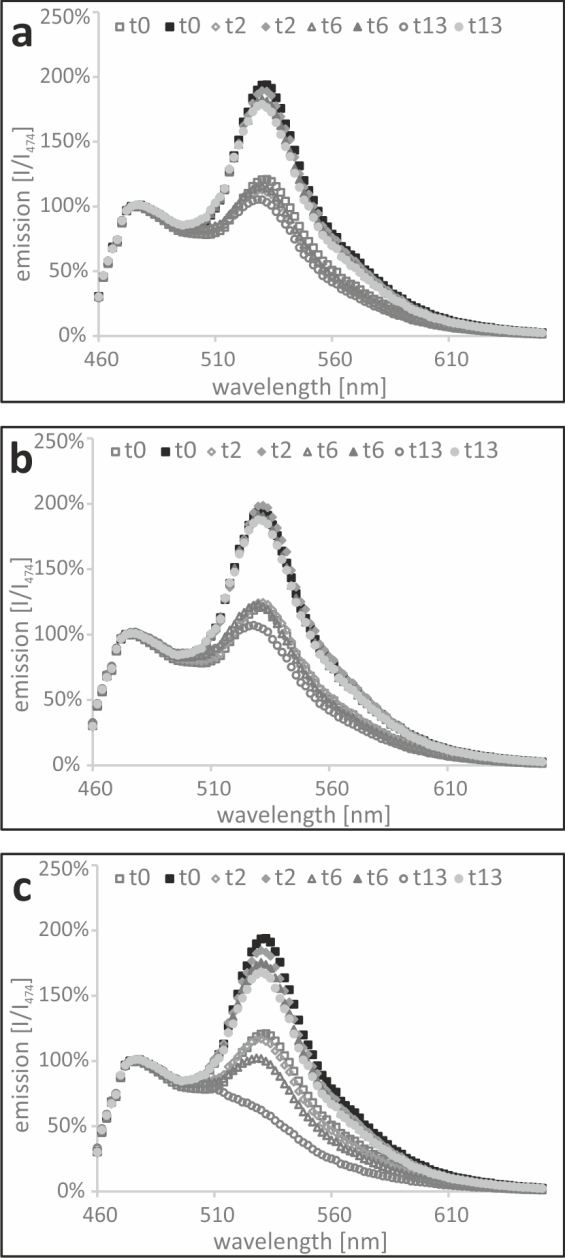


Figure S4: Emission spectra of the immobilized Glu^[+Halo]^. Solid markers indicate presence of 100 mM glucose, empty markers indicate no glucose. Incubation of the sensor at (a) 4°C, (b) -20°C and (c) 25°C without shaking. At room temperature (c), the sensor lost its functionality after 6 days, while the sensor stayed fully functional for at least 13 days when stored at 4°C (a) and -20°C (b).


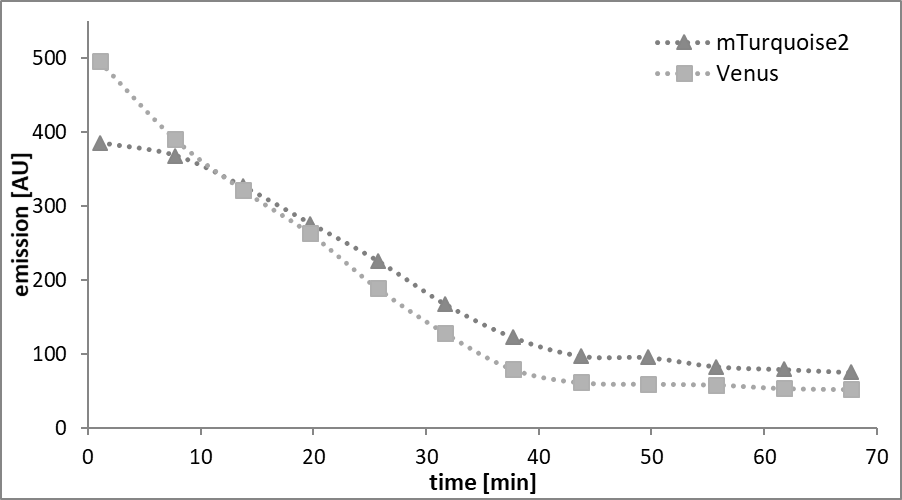


Figure S5: Stability of the Glu^[-]^ sensor in a micro bioreactor (BioLector, m2p-labs, Baesweiler, Germany) during shaking (1200 rpm, 30°C). Fluorescence emission of mTurquoise2 (λ_em_=486 nm) and Venus (λ_em_=532 nm) after excitation at λ_ex_= 430 nm were recorded in the BioLector cultivation device. The fluorescence signals of both fluorescence proteins incorporated in the Glu^[-]^ sensor started to decrease, immediately. After 35 minutes, the Glu^[-]^ sensor lost its functionality.


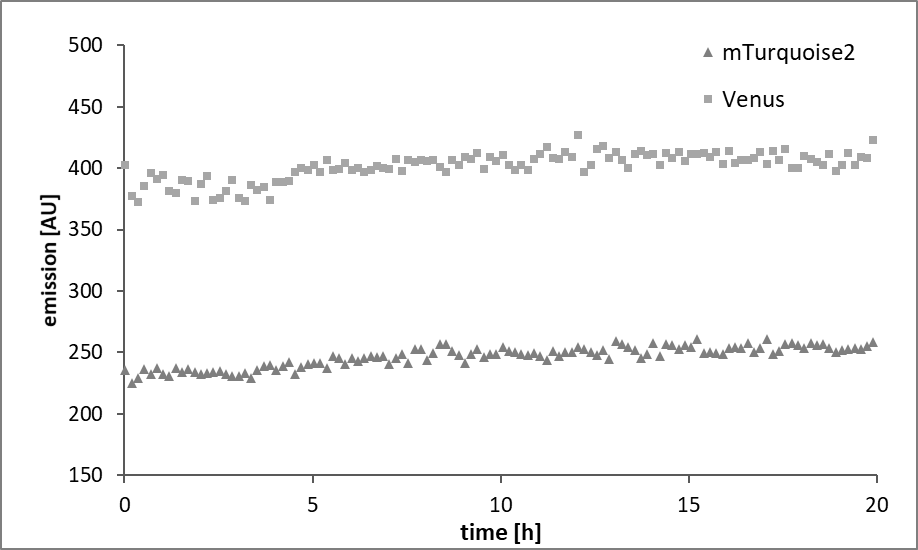


Figure S6: Stability of the immobilized Glu^[+Halo]^ sensor in a micro bioreactor (BioLector, m2p-labs, Baesweiler, Germany) during shaking (800 rpm, 30°C). Fluorescence emission of mTurquoise2 (λ_em_=486 nm) and Venus (λ_em_=532 nm) after excitation at λ_ex_= 430 nm were recorded in the BioLector cultivation device. No sensor degradation could be noticed throughout the whole process of 20 hours.

# At-line measurement process scheme


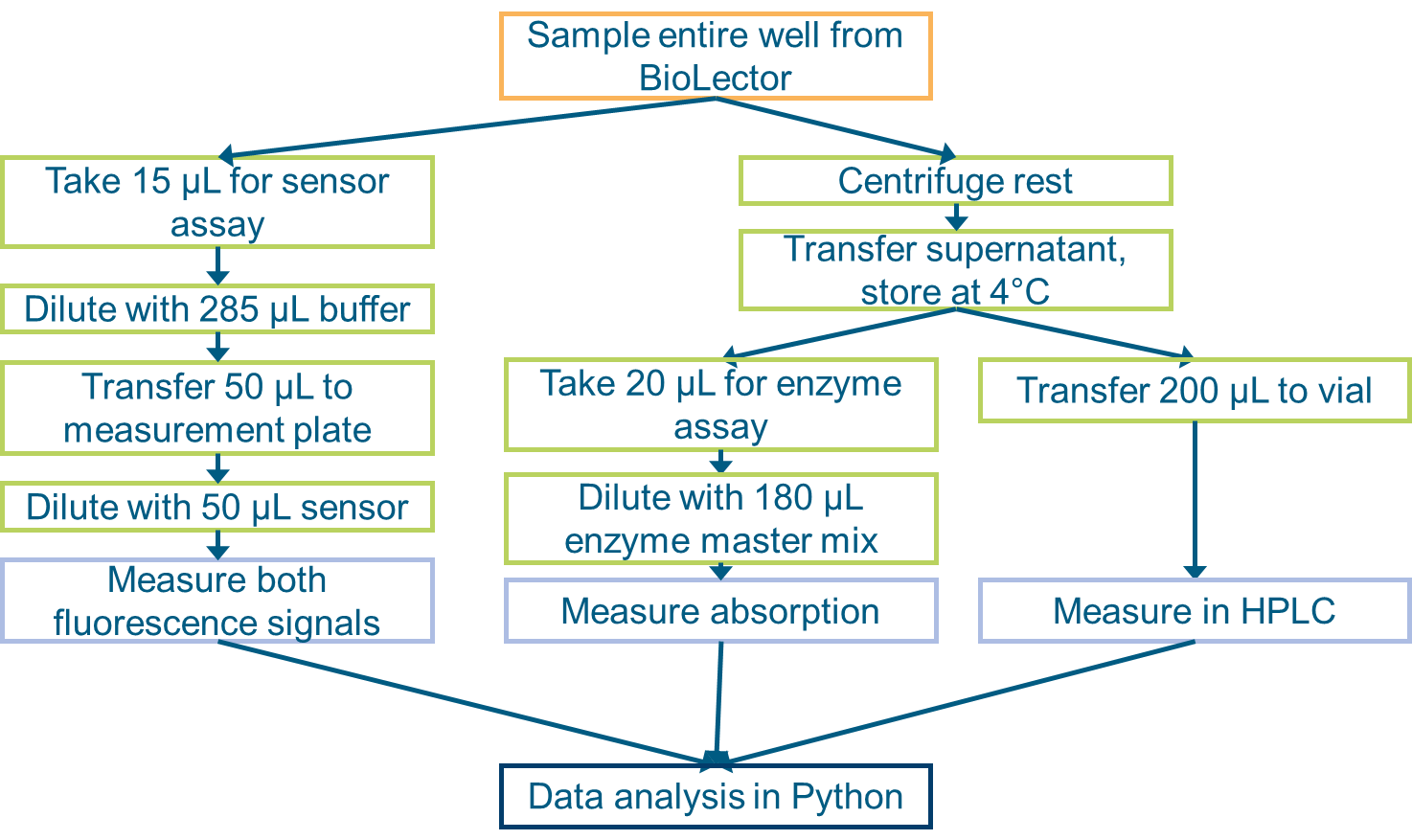


Figure S7: Schematic representation of the at-line process including the cultivation in a micro bioreactor (orange), sampling and processing steps (green) performed by the liquid handling system, comparative measurement of glucose concentrations via glucose biosensor (at-line), enzymatic assay, and HPLC (both offline) (blue) as well as the data analysis (black).

# Calibrations


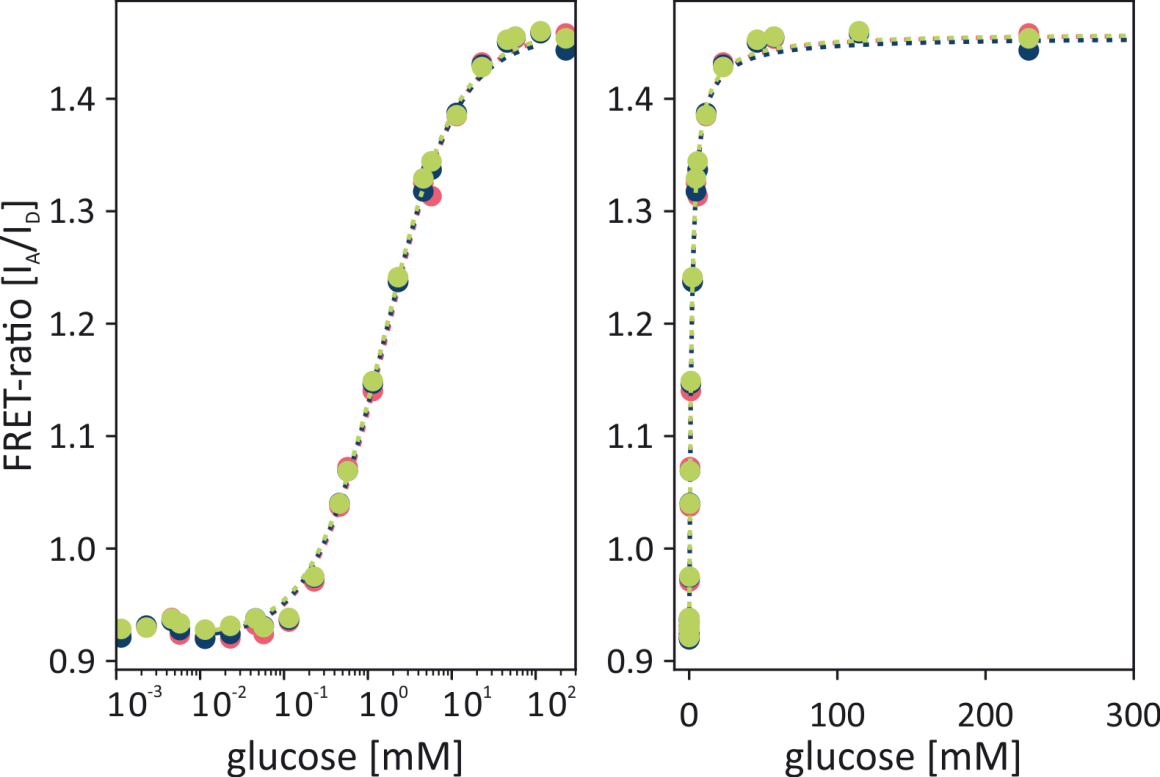


Figure S8: Calibration of the Glu^[-]^sensor used for the at-line quantification of glucose in a C. glutamicum ATCC 1332 cultivation using CGXII medium. Fitting of the data points according to equation 2 (main paper) resulted in an apparent K_d_ of 1.5 mM, R_0_ of 0.92, R_sat_ of 1.46, and ΔR of 58%; the red, green and blue data points represent three analytical replicates, each data point represents the mean of 5 technical replicates. The standard deviation (SD) is too small to be visible in this presentation.


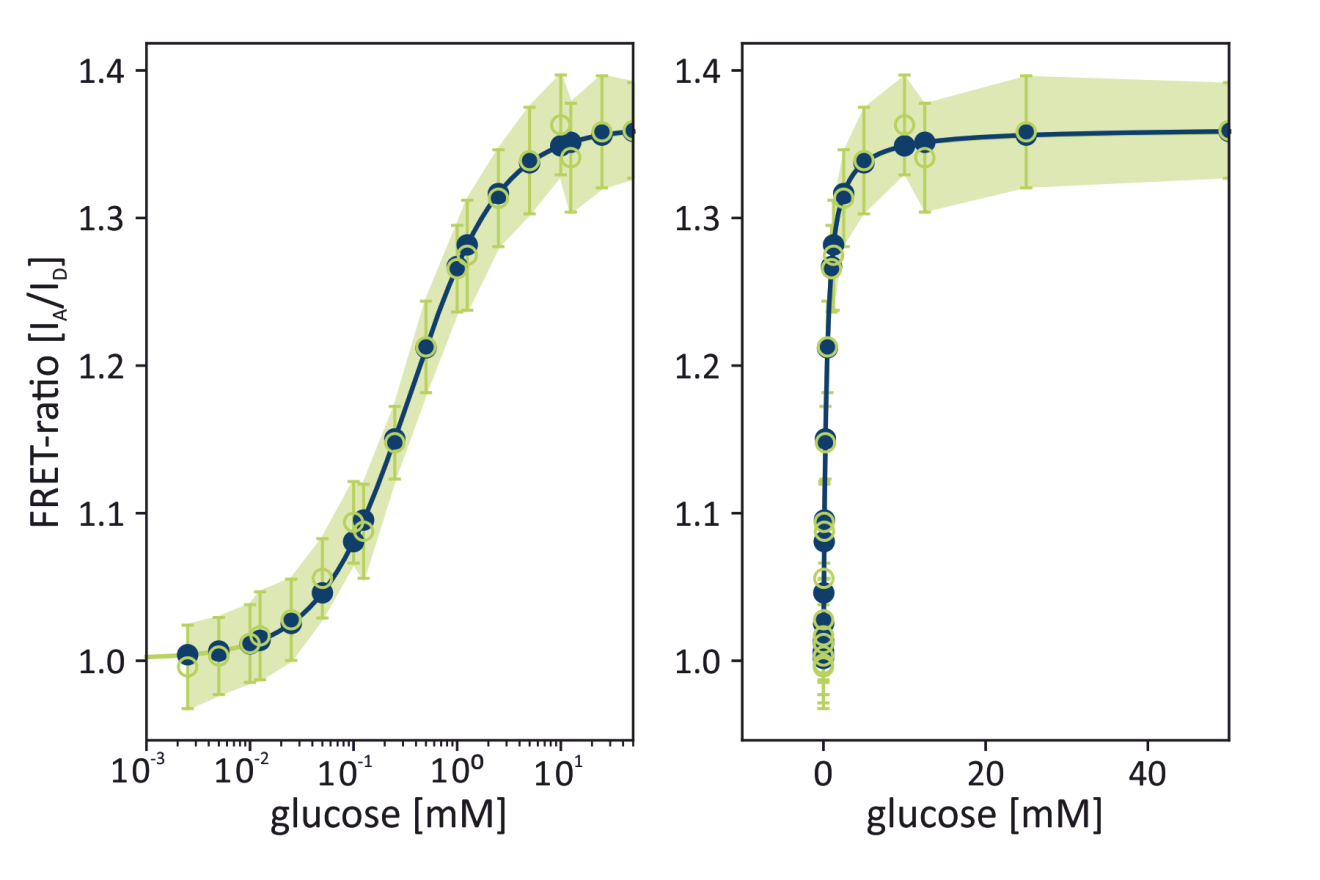


Figure S9: Calibration of the immobilized Glu^[+Halo]^ biosensor used for the online quantification of the glucose consumption of an E. coli K12 strain in M9 medium. In green, each data point resembles the mean ratio recorded over 20 hours within the BioLector. The filled area marks the standard deviation (SD) to demonstrate the noise of the signal, mainly caused by the shaking of the BioLector. Additionally the calibration curve is described by a saturation kinetic equation whose parameters are fitted to the measured calibration data by minimizing the sum of squares (blue). This resulted in an apparent K_d_ of 0.4 ± 0.1 mM, R_0_ of 1.0, R_sat_ of 1.36, and ΔR of ~36 % according to equation (2).


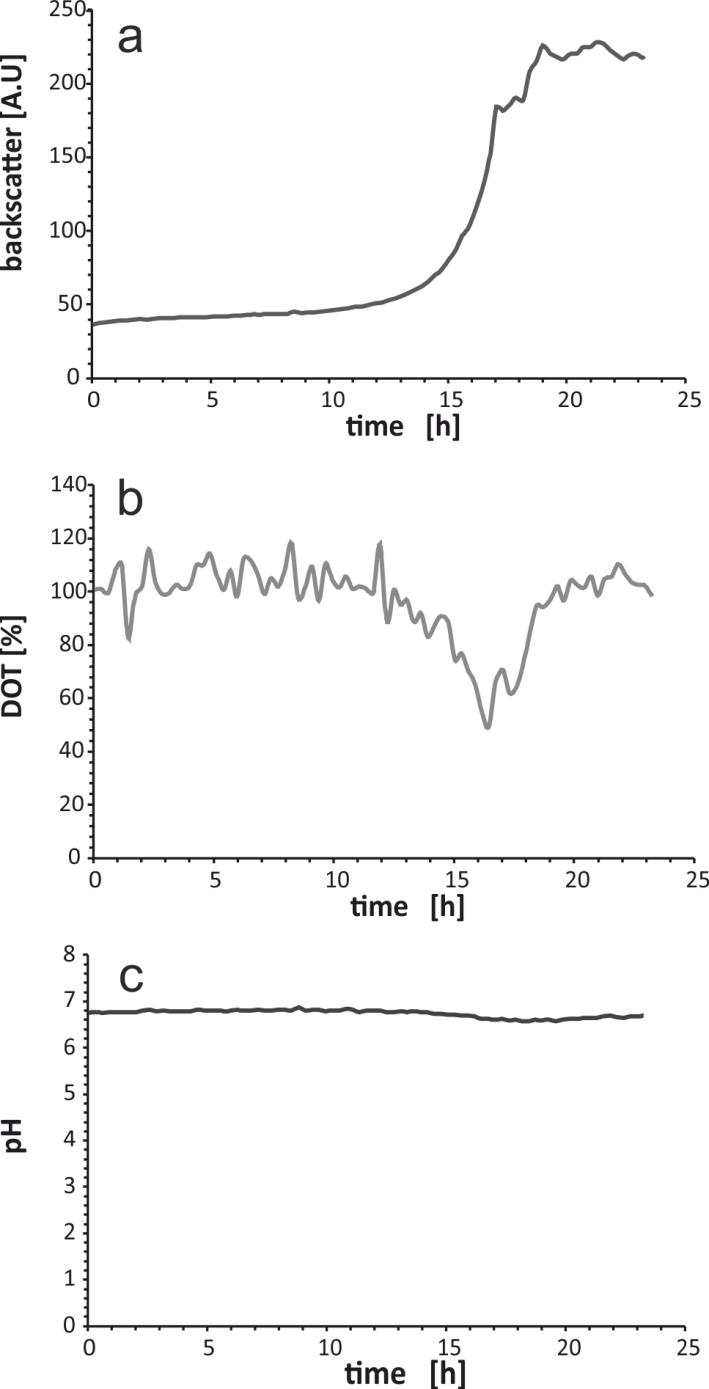


Figure S10: Additional process data from an E. coli MG1655 cultivation in M9 media with 5 g/L D-glucose. The cultivation was performed at 30°C and 900 rpm. Biomass formation (backscatter, a), oxygen availability (DOT, b), and pH (c) were monitored over 24 hours. The cultivation did not show signs of oxygen limitations (DOT> 40%) or a pronounced shift in pH.
